# Sexual assault experience, depression, and heavy substance use among German adults: an exploratory mediation analysis

**DOI:** 10.1186/s12889-025-22117-4

**Published:** 2025-03-10

**Authors:** Matthias Hans Belau, Christian Wiessner, Susanne Sehner, Arne Dekker, Peer Briken

**Affiliations:** 1 Institute of Medical Biometry and Epidemiology, University Medical Centre Hamburg-Eppendorf, Martinistr. 52, Hamburg, 20246 Germany; 2 Institute of Sex Research, Sexual Medicine, and Forensic Psychiatry, University Medical Centre Hamburg-Eppendorf, Hamburg, Germany

**Keywords:** Sexual assault, Substance use, Mental health, Depression, Mediation analysis, Germany

## Abstract

**Background:**

The experience of sexual assault may be associated with numerous adverse outcomes, including depressive disorders and heavy substance use. We aimed to examine the relationship between heavy substance use and depression in victims of sexual assault.

**Methods:**

We used nationally representative data from the German Health and Sexuality Survey (GeSiD) with *N* = 4,955 women and men aged 18–75 years. We assessed (i) the potential effect of sexual assault experience on depression mediated through hazardous alcohol, heavy tobacco, and frequent cannabis use and (ii) sexual assault experience on heavy substance use mediated through depression using logistic regression analysis to estimate proportion mediated (PM).

**Results:**

We found some evidence of mediation between sexual assault as a lifetime event and depression by heavy tobacco use (PM = 1.6%) and frequent cannabis use (PM = 14.7%) among women. We also observed mediation by hazardous alcohol use (PM = 35.5%) and heavy tobacco use (PM = 48.6%) among men who experienced childhood sexual assault. Focusing on depression as a potential mediator, we found some evidence of mediation between sexual assault as a lifetime event and heavy tobacco use among women (PM = 17.6%) and men (PM = 13.3%), and between sexual assault as a lifetime event and frequent cannabis use (PM = 26.9%) among women.

**Conclusions:**

Our findings suggest that public health specialists, clinicians, and therapists should develop early interventions to prevent addiction and the development of depression after experiencing sexual assault.

**Supplementary Information:**

The online version contains supplementary material available at 10.1186/s12889-025-22117-4.

## Background

Sexual violence is a widespread and serious public health problem [1]. It refers to any sexual assault, including penetrative sexual violence (rape) and non-penetrative sexual violence [2]. Findings from the Global Burden of Disease study suggest that the prevalence of sexual violence is higher among women than men [3]. In Germany, 13–15% of women and 3–7% of men become victims of sexual violence in their lifetime, many of them in childhood [4–6]. Possible consequences of sexual violence include serious psychological disorders, as well as physical, sexual, and reproductive health problems [1]. The relationship between child sexual violence and subsequent mental disorders is well established [7, 8]. Evidence also suggests that depressive disorders are particularly prevalent among those who have experienced sexual assault and rape [8, 9]. A depressive disorder is defined as any mood disorder that typically presents with symptoms such as sadness, emptiness, or irritability, adversely impacting emotional, cognitive, and physical functioning, thereby significantly impairing daily life [10]. Several studies showed that depression is associated with increased use of substances such as alcohol, tobacco, and cannabis [11–13]. A recent meta-analysis found that victims of sexual assault and rape are more likely to suffer from substance use disorders [9]. The association between childhood abuse and substance use disorders later in life is well established [14, 15]. A substance use disorder is defined as a cluster of psychological, behavioral, and cognitive symptoms associated with continued substance use despite the presence of substance-related problems, distress, and/or impairment [10].

So far, most research has focused on depressive disorders and substance use disorders impacted by sexual assault [9, 16]. Depression and substance use disorders often co-occur in victims of sexual assault [9, 17]. However, less is known about the causal mechanism between sexual assault, heavy substance use, and depression over the life course. An earlier review found that sexual abuse in childhood is associated with substance use in adulthood [18]. Large genome-wide association studies have shown that substance use disorders have a positive genetic association with major depression [19, 20]. Some previous studies have suggested a relationship between sexual assault and substance use and abuse that is mediated by depression [21, 22]. The prevailing hypothesis is that substance use behaviors are strategies to cope with psychological distress or depression related to sexual assault, often discussed as the ‘self-medication’ hypothesis. In contrast to these other studies, several meta-analyses suggest that the mechanism by which heavy substance use causes depression is enhanced and more pervasive [11–13]. However, whether such associations reflect causality in sexual assault victims remains unknown, and the evidence gaps need to be filled. To disentangle the relationship between sexual assault experience (SAE) as an exposure and both depression and heavy substance use as a potential mediator or outcome, mediation analyses were indicated. The German Health and Sexuality Survey (GeSiD) is the first nationwide population-based sex survey in Germany to include questions about sexual violence and health. Understanding the relationship between heavy substance use and depression in victims of sexual assault is critical. A better understanding of how these two issues interact can help to develop more targeted and effective interventions, ultimately leading to improved outcomes. In addition, awareness of the interplay between substance use and depression among sexual assault survivors can inform policy and advocacy efforts aimed at improving support services, funding research, and developing trauma-informed care in health care settings. Therefore, we aimed to examine (i) the potential effect of SAE on depression mediated through heavy substance use and (ii) SAE on heavy substance use mediated through depression among German adults in a nationally representative sample.

## Materials and methods

### Study design and participants

**Fig. 1:**
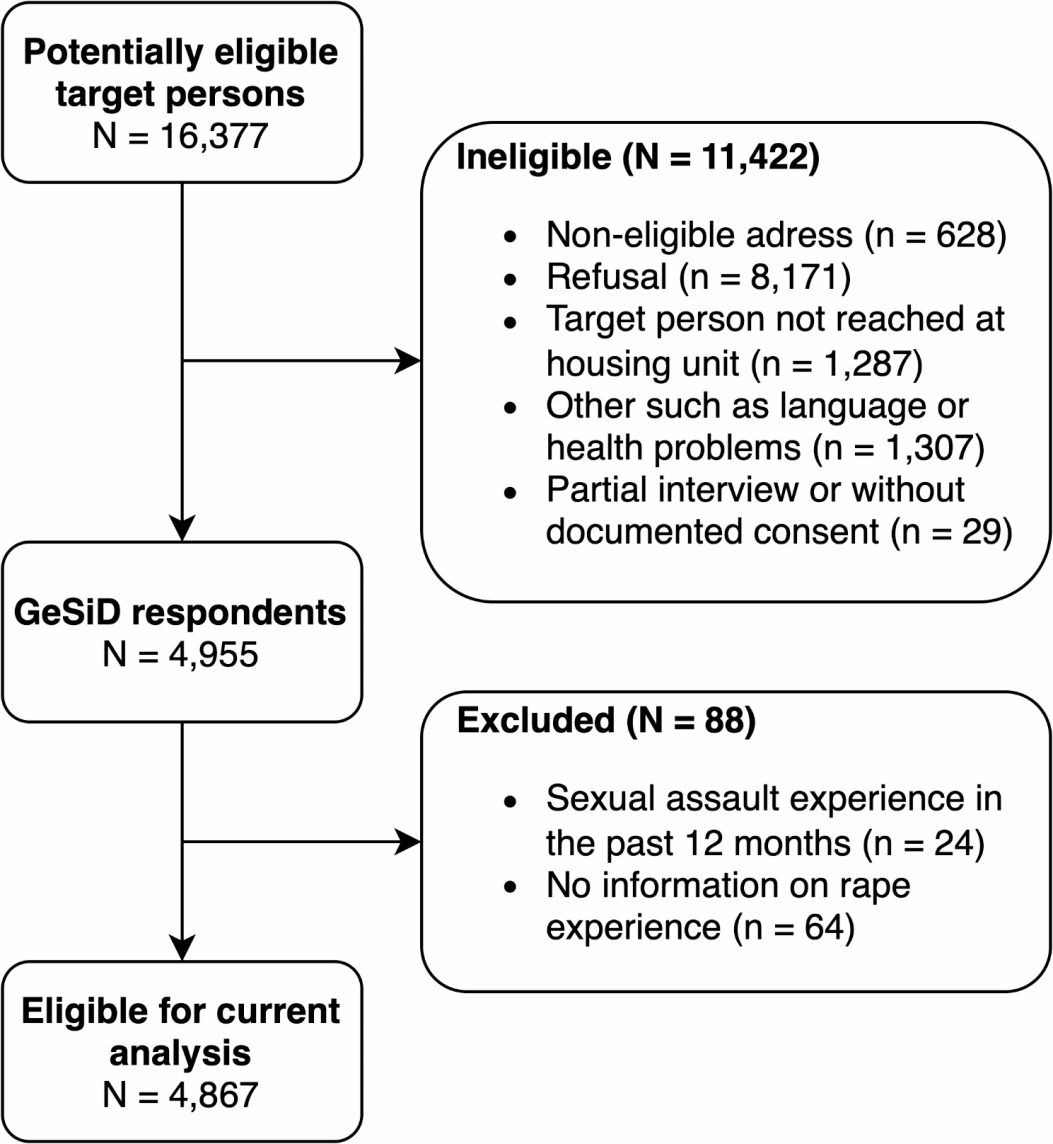
Respondent flow-chart

Between October 2018 and September 2019, we interviewed women and men aged 18 to 75 years who were residents of Germany. The sample was selected through a two-stage selection process. The initial stage of the sampling process involved the random selection of 178 municipalities, amounting to 200 sampling points (regional clusters spread across the municipalities). The municipal sample was conducted as an area sample with a four-dimensional matrix (federal state x region x district x BIK, a nationwide German spatial classification system representing the urban-rural relationship), using the number of persons between the ages of 18 and 75 years as a stratification weight. Age stratification ensures that different age groups are adequately represented in the target sample. Subsequently, the registration offices of the selected municipalities were requested to provide a random sample of individuals according to a three-dimensional matrix (federal state x age group x sex), with an identical number of addresses for each sample point. In the event that a municipality was allocated more than one sample point (which was predominantly the case for larger cities), additional addresses were provided. A total of *N* = 16,377 individuals were assessed for eligibility. Of these, *N* = 4,955 signed informed consent forms and completed the survey, representing a response rate of 30.2% (AAPOR response rate 4 [23], adapted for the German context [24]). Computer-assisted personal interviews (CAPI) conducted by trained interviewers from Kantar GmbH using a standardized questionnaire were used for most questions, while computer-assisted self-administered interviews (CASI) using a standardized questionnaire were employed for more sensitive questions. The questionnaire covered a wide range of topics to sexual and general health, as well as the respondents’ current life circumstances. The questionnaire was developed based on an extensive review of existing research on similar topics [25, 26]. The World Health Organization’s indicators of sexual health [27] were considered and modified to align with the specific context of the German survey. Respondents without information on the exposure to sexual assault (*n* = 64), and those reporting exposure to sexual assault in the past 12 months (*n* = 24) were excluded from the analysis (see Fig. 1). The final analytic sample was 4,867 women and men for the mediation analyses. This study was performed in line with the principles of the Declaration of Helsinki. Written informed consent was obtained from all study participants. Approval was granted by the Ethical Board of the State Psychotherapy Chamber in Hamburg (reference: 07/2018-PTK-HH) before the data was collected to ensure ethical and data protection guidelines. Further details of the study design, sample design, recruitment and methods used, as well as results pertaining to non-response follow-up analysis and representativeness of the GeSiD sample, can be found in other publications [28, 29].

### Variables

#### Exposure

In the computer-assisted self-interview section of the questionnaire, participants were asked ‘Has anyone ever had or tried to have oral, anal, or vaginal sex (intercourse) with you against your will’, which was used to define lifetime SAE (no, yes). Participants who answered ‘yes’ were then asked ‘How old were you when this first happened to you’. Participants who reported SAE before the age of 14 were also asked ‘Was this person at least five years older than you’, which was used to define childhood SAE in line with a previous study [6].

#### Potential mediators and outcome

We further asked participants about their use of alcohol, tobacco, and cannabis in the computer-assisted self-interview section of the questionnaire. We defined hazardous alcohol consumption as an average consumption of 14 or more drinks per week for women (or ≥5 drinks per occasion at least 3 times per week), and 21 or more drinks per week for men (or ≥7 drinks per occasion at least 3 times per week), according to the usual World Health Organization criteria [30].

We also asked participants whether they had ever used tobacco on a regular basis and, if so, whether they had smoked in the past 12 months and how many cigarettes they usually smoked per day. Respondents who smoked more than 20 cigarettes per day (a pack) were classified as heavy smokers [31].

Participants were then asked ‘Have you used cannabis (marijuana, weed, hashish) in the last 12 months? Participants who answered ‘yes’ were then asked ‘How often have you used cannabis (marijuana, weed, hashish) in the last 12 months’. Participants who reported using cannabis on a daily or almost daily basis were defined as frequent users, according to the European Monitoring Centre for Drugs and Drug Addiction [32].

Finally, participants were asked if they had received treatment for depression in the past 12 months. The responses were categorized as depression in the past 12 months vs. no depression in the past 12 months.

### Covariates

In the computer-assisted personal interview, we obtained data on all covariates to adjust for known confounders. Sociodemographic factors included age at the interview, sex (female, male) according to the registration office [29], and education (primary, secondary, tertiary). The age variable was categorized into six age groups (18–25, 26–35, 36–45, 46–55, 56–65, and 66–75) due to violation of linearity in logistic regression analyses, as detailed below.

### Statistical analysis

**Fig. 2:**
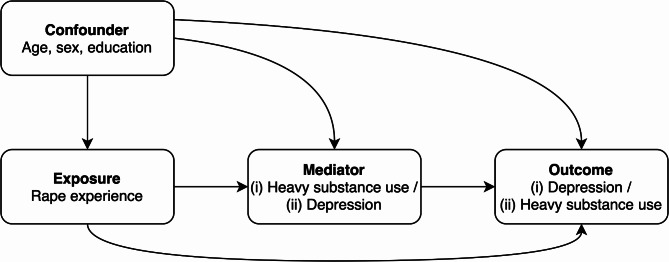
Directed acyclic graph

All analyses were conducted on a sex-specific basis, with women and men analyzed separately. This is because differences in the prevalence of SAE [4–6], depression [33], and substance use [34] between men and women are well documented. Descriptive statistics were calculated in the form of prevalence estimates with 95% confidence intervals (CIs) for the total number of respondents with valid data. Individuals with incomplete data were excluded from the respective analyses. The number of missing values was < 5%. The differences in prevalence estimates were deemed statistically significant when the 95% CIs did not overlap. It is acknowledged that this method is conservative and that the failure to reject the null hypothesis does not necessarily indicate the rejection of the null hypothesis through statistical tests [35]. We used causal mediation methods [36] to determine (i) the effect of SAE (more than 12 months ago) on depression (within the last 12 months) that was mediated by the potential heavy substance use (within the last 12 months) mediators, and (ii) the effect of SAE (more than 12 months ago) on heavy substance use (within the last 12 months) that was mediated by the potential depression (within the last 12 months) mediator. Given the ambiguity of the temporal sequence of substance use and depression, as the GeSiD survey aimed to assess the 12-month prevalence of both, recursive models were assumed to test the mediation hypotheses. The recursive model assumes that once a variable such as heavy substance use is influenced by another variable such as depression, it will not return to influence the first variable within the scope of the analysis. This is useful when examining causal pathways in exploratory research and allows for clearer interpretations of the pathways being analyzed. Cases presenting a missing value for at least one of the modeling variables were excluded from analyses (listwise deletion). We are aware that this method may reduce the statistical power of the mediation analysis [37]. However, we could not find a specific characteristic for incomplete responses and the number of missing values was < 5%. A representation of the assumed causal models can be found in Fig. 2. The mediators were assessed through multivariable logistic regression models of the outcome and the mediators, and results were then combined to estimate the natural direct effect (NDE), the natural indirect effect (NIE), and the total effect (TE) [38]. In this counterfactual-based approach, the NDE captures the effect of the exposure on the outcome that would remain if the pathway from the exposure to the mediator were to be eliminated. The NIE, on the other hand, captures the effect of the exposure on the outcome that occurs by altering the mediator. The TE decomposes into the NDE and NIE. We computed odds ratios (ORs) and 95% percentile bootstrap CIs from 1,000 bootstrap samples. NIEs were considered significant if the 95% CI did not contain one. In addition, proportion mediated (PM) defined as the ratio of the NIE to the TE was calculated [36]. On the log scale, this is $\:\text{log}\left({OR}^{NIE}\right)/\:\text{l}\text{o}\text{g}\left({OR}^{TE}\right)$. We first examined respondents who reported a lifetime SAE versus no SAE, and then in a subsequent analysis, we compared those who reported a childhood SAE with participants who had no SAE. In a sensitivity analysis, we examined respondents who reported more than one lifetime and childhood SAE versus no SAE because some studies suggest a higher risk of mental illness and substance abuse among those with multiple SAEs [14, 39]. We were unable to examine more than one SAE versus one SAE due to the small number of events. We did all analyses with the survey procedures (SVY) in Stata MP (version 18), which incorporated the weighting, clustering, and stratification of the GeSiD dataset [29].

## Results

Study population characteristics (*N* = 4,867) by sex are shown in Table 1. SAE was reported by 14.0% of all women and 2.7% of all men. Almost a fifth (19.3%) of women and about a third (33.7%) of men who reported rape had been victims as children. The mean and median age (first quartile (Q1), third quartile (Q3)) at the first occurrence of SAE was 18.1 and 17 years (Q1 = 14, Q3 = 21) for women and 17.1 and 16 years (Q1 = 12, Q3 = 20) for men. The weighted proportion of respondents who reported depression in the past 12 months among all respondents was 8.1% (95% CI = [7.3, 9.1%]). This figure was higher for women (10.8%) than for men (5.6%), indicating a greater prevalence of depression among the female population. Men were more likely than women to report heavy tobacco use (12.3% vs.7.8%) and frequent cannabis use (2.6% vs.1.0%), while the prevalence of hazardous alcohol use in the past 12 months was similarly low (0.9% vs. 0.7%).

**Table 1:** Prevalence of sexual assault experience, heavy substance use, depression, and sociodemographic (*N* = 4,867)

	Women				Men			
	n = 2,563				n = 2,304			
	**%**	**95% CI**			**%**	**95% CI**		
		**Lower**	**Upper**	**n**		**Lower**	**Upper**	**n**
**Age group**								
18–25	11.5	10.2	12.8	370	12.5	11.1	14	383
26–35	17.1	15.6	18.6	554	18	16.4	19.7	532
36–45	16.4	14.9	18	426	16.2	14.6	18	374
46–55	21.3	19.5	23.3	488	21.8	19.8	23.9	358
56–65	19.2	17.5	21	488	18.7	16.9	20.7	373
66–75	14.3	12.6	16.1	237	12.6	11	14.3	284
**Education**								
High	36.5	33.9	39.3	1,243	36.6	33.9	39.4	1,108
Medium	35.2	32.9	37.5	872	28.9	26.5	31.4	664
Low	28.1	25.4	31	448	34.4	31.5	37.3	532
**SAE** (lifetime event)								
No	85.9	84.3	87.4	2,189	97.3	96.3	97.9	2,239
Yes	14	12.6	15.6	374	2.7	2	3.6	65
**SAE** (childhood event)								
No	96.8	95.8	97.5	2,189	99.1	98.6	99.4	2,239
Yes	3.1	2.4	4.1	72	0.8	0.5	1.3	20
*Missing values*	-	-	-	302	-	-	-	45
**Hazardous alcohol use**								
No	99.2	98.8	99.5	2,425	99	98.5	99.3	2,209
Yes	0.7	0.4	1.2	19	0.9	0.6	1.4	23
*Missing values*	-	-	-	119	-	-	-	72
**Heavy tobacco use**								
No	92.1	90.8	93.3	2,394	87.6	85.8	89.3	2,056
Yes	7.8	6.6	9.2	162	12.3	10.6	14.2	238
*Missing values*	-	-	-	7	-	-	-	10
**Frequent cannabis use**								
No	98.9	98.3	99.3	2,528	97.4	96.6	98	2,232
Yes	1	0.7	1.6	31	2.6	1.9	3.3	67
*Missing values*	-	-	-	4	-	-	-	5
**Depression**								
No	89.2	87.7	90.6	2,301	94.3	93.1	95.3	2,181
Yes	10.7	9.4	12.2	262	5.6	4.6	6.8	123

%: quantity (weighted), CI: confidence interval (weighted), n: quantity (unweighted), SAE: sexual assault experience

Prevalence rates of depression by sociodemographic, SAE, and heavy substance use characteristics can be found in Supplementary Material S1. The prevalence of depression was higher among women and men who reported lifetime SAE than among those who did not report SAE. Women who reported childhood SAE were four times more likely to report depression than those who did not report childhood SAE. In addition, women who reported heavy tobacco use and frequent cannabis use had a higher prevalence of depression. In contrast, no differences were observed in the prevalence of depression among men with different patterns of substance use.

Supplementary Material S2 provides the prevalence rates of heavy substance use by sociodemographic, SAE, and depression characteristics. Hazardous alcohol use was highest among men who reported childhood SAE. Women who reported depression, were twice as likely to report heavy tobacco use compared as those who did not report depression, whereas the prevalence of heavy tobacco use among men varied by childhood SAE. The prevalence of frequent cannabis use was again highest among those reporting SAE and depression.

The mediation results between SAE as a lifetime event and depression through heavy substance use stratified by sex are shown in Table 2. We found some evidence for mediation between SAE as a lifetime event and depression among women through heavy tobacco use (PM = 1.6%; NIE = 1.02 (95% CI = [1.01, 1.05])) and frequent cannabis use (PM = 14.7%; NIE = 1.23 (95% CI = [1.01, 1.85])). In addition, we found some evidence for mediation between SAE as a childhood event and depression among men through hazardous alcohol use (PM = 35.5%; NIE = 1.16 (95% CI = [1.01, 2.17])) and heavy tobacco use (PM = 48.6%; NIE = 1.21 (95% CI = [1.02, 1.72])).

**Table 2:** Mediation analyses between sexual assault experience (lifetime, childhood event) and depression by sex, adjusted for age, and education (*N* = 4,867)

			Lifetime event ^a^								Childhood event ^b^						
**Depression**			**Women**				**Men**				**Women**				**Men**		
			**OR**	**95% BCI**			**OR**	**95% BCI**			**OR**	**95% BCI**			**OR**	**95% BCI**	
				**Lower**	**Upper**			**Lower**	**Upper**			**Lower**	**Upper**			**Lower**	**Upper**
*Mediator*																	
Hazardous alcohol use	Total effect		3.36	2.37	4.60		3.59	1.34	7.1		5.23	2.80	9.02		1.52	0.72	3.86
	Natural direct effect		3.36	2.37	4.60		3.51	1.31	6.97		5.23	2.80	9.02		1.31	0.64	2.67
	Natural indirect effect		1.00	0.99	1.01		1.02	0.98	1.17		1.00	1.00	1.00		1.16	1.01	2.17
*Mediator*																	
Heavy tobacco use	Total effect		3.43	2.46	4.76		3.60	1.20	7.83		5.55	2.86	10.30		1.48	0.99	3.46
	Natural direct effect		3.38	2.40	4.68		3.39	1.12	7.53		5.46	2.77	10.16		1.22	0.77	2.79
	Natural indirect effect		1.02	1.01	1.05		1.06	0.99	1.19		1.01	0.99	1.07		1.21	1.02	1.72
*Mediator*																	
Frequent cannabis use	Total effect		4.09	2.74	6.79		3.62	1.25	7.15		7.55	3.19	13.9		1.57	1.02	7.07
	Natural direct effect		3.31	2.32	4.69		3.66	1.26	7.17		5.70	3.04	11.06		1.57	1.02	7.07
	Natural indirect effect		1.23	1.01	1.85		1.00	0.95	1.01		1.32	0.98	1.91		1.00	1.00	1.00

OR, odds ratio; BCI, confidence interval from 1,000 bootstrap samples, ^a^ Sample size ranged from 2,444 to 2,559 in women and from 2,2232 to 2,299 in men, ^b^ Sample size ranged from 2,168 to 2,274 in women and from 2,195 to 2,261 in men

Brief description:

There is an association between SAE as a lifetime event and depression among women mediated through heavy tobacco use and frequent cannabis use. In addition, there is an association between SAE as a childhood event and depression among men mediated through hazardous alcohol use and heavy tobacco use

In sensitivity analyses among those who reported SAE more than once versus no SAE, we found a higher PM and slightly stronger effect for mediation among women between SAE as a lifetime event and depression through frequent cannabis use (PM = 16.0%; NIE = 1.39 (95% CI = [1.07, 3.12])) and among men between SAE as a childhood event and depression through heavy tobacco use (PM = 27.6%; NIE = 1.24 (95% CI = [1.02, 1.80])) (see Supplementary Material S3).

We also observed some evidence for mediation between SAE and heavy substance use through depression (Table 3). We observed associations between SAE as a lifetime event and heavy tobacco use through depression among women (PM = 17.6%; NIE = 1.10 (95% CI = [1.02, 1.25])) and men (PM = 13.3%; NIE = 1.14 (95% CI = [1.01, 1.53])), and between SAE as a lifetime event and frequent cannabis use through depression among women (PM = 26.9%; NIE = 1.45 (95% CI = [1.09, 2.00])).

**Table 3:** Mediation analyses between sexual assault experience (lifetime, childhood event) and heavy substance use by sex, adjusted for age, and education (*N* = 4,867)

			Lifetime event ^a^								Childhood event ^b^						
**Hazardous alcohol use**			**Women**				**Men**				**Women**				**Men**		
			**OR**	**95% BCI**			**OR**	**95% BCI**			**OR**	**95% BCI**			**OR**	**95% BCI**	
				**Lower**	**Upper**			**Lower**	**Upper**			**Lower**	**Upper**			**Lower**	**Upper**
*Mediator*																	
Depression	Total effect		1.79	0.30	5.69		5.30	1.60	16.74		0.86	0.75	1.29		20.44	7.96	72.27
	Natural direct effect		1.91	0.30	6.10		4.63	1.59	12.58		1.00	1.00	1.00		19.46	7.47	64.08
	Natural indirect effect		0.93	0.86	1.15		1.14	0.93	1.72		0.86	0.75	1.29		1.05	0.99	2.00
**Heavy tobacco use**			**Women**				**Men**				**Women**				**Men**		
			**OR**	**95% BCI**			**OR**	**95% BCI**			**OR**	**95% BCI**			**OR**	**95% BCI**	
				**Lower**	**Upper**			**Lower**	**Upper**			**Lower**	**Upper**			**Lower**	**Upper**
*Mediator*																	
Depression	Total effect		1.72	1.01	2.65		2.67	1.09	5.16		2.14	0.83	4.22		5.86	2.16	14.86
	Natural direct effect		1.55	0.89	2.42		2.33	0.99	4.71		1.84	0.68	3.84		5.61	1.97	14.16
	Natural indirect effect		1.10	1.02	1.25		1.14	1.01	1.53		1.16	0.96	1.51		1.04	0.99	1.26
**Frequent cannabis use**			**Women**				**Men**				**Women**				**Men**		
			**OR**	**95% BCI**			**OR**	**95% BCI**			**OR**	**95% BCI**			**OR**	**95% BCI**	
				**Lower**	**Upper**			**Lower**	**Upper**			**Lower**	**Upper**			**Lower**	**Upper**
*Mediator*																	
Depression	Total effect		3.99	1.67	9.92		0.46	0.32	1.86		3.71	0.53	12.95		1.06	0.97	1.71
	Natural direct effect		2.74	1.05	7.18		0.36	0.24	1.51		1.99	0.26	10.50		1.00	1.00	1.00
	Natural indirect effect		1.45	1.09	2.00		1.27	0.94	1.74		1.85	0.88	3.07		1.06	0.97	1.71

OR, odds ratio; BCI, confidence interval from 1,000 bootstrap samples, ^a^ Sample size was 2,503 in women and 2,304 in men, ^b^ Sample size was 2,277 in women and 2,267 in men

Brief description:

There is an association between SAE as a lifetime event and heavy tobacco use mediated through depression among women and men, and between SAE as a lifetime event and frequent cannabis use mediated through depression among women

In sensitivity analyses, we found a higher PM and slightly stronger effect for mediation among women between SAE as a lifetime event and heavy tobacco use through depression (PM = 28.8%; NIE = 1.21 (95% CI = [1.03, 1.52])) and between SAE as a lifetime event and frequent cannabis use through depression (PM = 38.8%, NIE = 1.72 (95% CI = [1.07, 3.01])) (see Supplementary Material S4).

## Discussion

In this large, nationally representative study of women and men aged 18 to 75 years, we found some evidence that hazardous alcohol, heavy tobacco, and frequent cannabis use mediated the association between SAE and depression. In fact, cannabis was a mediator for women, alcohol for men, but tobacco was a mediator for both sexes. Sensitivity analysis focusing on SAE more than once showed that cannabis was a mediator for women and tobacco for men with slightly stronger effects compared to SAE at least once, but the 95% CIs overlap. Tobacco use is a well-known coping mechanism for dealing with trauma and stress in women and men [40]. Research also suggests that men who have experienced childhood sexual assault have high rates of hazardous drinking and possible dependence [41], and that sexual victimization does not make a substantial independent contribution to heavy drinking among women in the general population [42]. However, women may use cannabis for its anxiolytic properties, particularly in response to traumatic experiences [43]. In addition, we observed some evidence of an association between SAE and heavy tobacco or frequent cannabis use mediated through depression. These effects were demonstrated only for women in the sensitivity analysis, with slightly stronger effects compared to SAE at least once, but the 95% CIs overlap. Additionally, among women, a bidirectional relationship between depression and cannabis or tobacco use was identified, which is to some extent consistent with the extant literature [44–46].

When comparing the results of our study with those of previous studies, there are some discrepancies. We identified only one small study (204 Latina women aged 18 to 34 years) that focused on potential mediators of lifetime substance use through which sexual abuse might affect depression, but no mediation effect was found [47]. A second study focused on the mediating effect of substance use coping between childhood sexual abuse and depressive symptoms among female and male methamphetamine users in South Africa [48]. On the other hand, one study suggested an association between sexual assault and hazardous alcohol use among women, mediated by depression [21]. Other studies focused on the mediating effect of depression between childhood abuse and harmful substance use among adults aged no more than 24 years [49] and criminal offenders [50]. In contrast to these other studies, meta-analyses showed that the mechanism by which heavy substance use causes depression is stronger and more prevalent [11–13], and the strength of the association is a Bradford Hill criterion for establishing causality [51], which refers to how strongly an exposure is related to the mediator and outcome.

Our results on the TE are consistent with past studies suggesting an association between lifetime SAE and depression in women and men. A meta-analysis found that lifetime experience of sexual assault was associated with a 3.10 to 3.44 times higher risk of depression than that among those who had not experienced sexual assault [9]. On the other hand, lifetime sexual assault was associated with 1.75 times the risk of alcohol use disorders and 3.43 times the risk of drug use disorders [9]. We were somewhat surprised that the NDE between childhood SAE and depression was about 4 times larger in women. An umbrella review of the long-term health effects of child abuse found that the odds of developing depressive disorders were 3 times higher [52]. A possible reason for the discrepancy between the OR estimates for women and men may be sex differences in the relationship between trauma and psychopathology [53]. Evidence suggests that women may process trauma differently than men [54, 55], which may result in elevated rates of internalizing disorders. Additionally, men may be less inclined to disclose their trauma due to social stigma, which can influence the diagnostic process and the efficacy of treatment [55].

Additionally, heavy tobacco use and frequent cannabis use explained a high percentage of the association between lifetime SAE and depression in women, whereas hazardous alcohol use and heavy tobacco use explained a high percentage of the association between childhood SAE and depression in men. These findings suggest a possible need to tailor a heavy substance use prevention program to sexual assault victims. This could reduce about 15 to 30% of the possible effect of SAE on depression. Interrupting the pathway from childhood SAE to heavy substance use, especially in men, could prevent an even higher proportion of depression (about 50%). A similar picture, albeit with smaller estimated effect sizes, is seen for the association between SAE and heavy substance use that is mediated by depression.

A comparison of depression in GeSiD with a study using countrywide outpatient claims data from all residents with statutory health insurance in 2017 revealed that the 12-month prevalence estimates were lower for women and men in GeSiD [56]. In the German Health Update study (period, 04/2019-01/2020), the prevalence of depressive symptoms in the past two weeks was assessed by self-report of participants using the 8-item Patient Health Questionnaire (PHQ-8). The results indicated that 9.8% of women and 8.5% of men met the criteria for depressive symptoms [57]. It is essential to recognize that comparisons between the prevalence estimates of depression in GeSiD and the prevalence of depression observed in other representative studies are influenced by discrepancies in sample composition and the use of different instruments. Furthermore, the data on depression in GeSiD were collected in response to a single question on receipt of treatment for depression, which may have led to misclassification of individuals with untreated depression. Additionally, it is probable that individuals with SAE and/or heavy substance use may have had difficulty or been unable to obtain treatment for depression. It is also likely that individuals with current depression (at the time of the survey) and severe cases are underrepresented in the GeSiD sample. Consequently, the prevalence estimates presented herein are likely to be conservative.

### Limitations and strengths

The findings of this study should also be considered in the context of the following limitations: First, our data on depression rely on answers to a single question, and differences may exist between women and men who self-report depression and those who meet the criteria for depression in a comprehensive standardized diagnostic interview [58]. Second, the measure of SAE was similarly collected through self-report and might suffer from underreporting or measurement error. Third, we did not distinguish between attempted and completed SAE events; however, even less severe forms of sexual harassment have been shown to have adverse effects on mental health [59, 60]. Fourth, the temporal order of the potential mediators and the outcome was not precisely defined, which limits the interpretation of the results. Heavy substance use may be an important mediator over longer periods; this study considered mediator and outcome variables that were likely to have occurred within the same year. We adopted recursive models in which SAE precedes heavy substance use or depression, and through this pathway influences depression or heavy substance use. It is important to note that depression may lead to heavy substance use and vice versa. Therefore, a longitudinal study following sexual assault victims and unexposed controls over time may be informative for further analysis. Fifth, the precision of the results may be insufficient due to small numbers of cases in some variables of interest, especially heavy alcohol and frequent cannabis use. Finally, we might not have accounted for all factors that could confound the relationship between SAE and depression. Potential confounders for which we could not adjust include the social network and its support, as well as information about post-traumatic stress disorder, antisocial behavior, and specific treatment experiences. Our study sample included predominantly women and men of European ancestry who were residents of Germany, so generalizing our findings to women and men of other ethnicities should be done with caution.

Despite these limitations, this study contributes to a better understanding of the relationship between depression and heavy substance use among women and men with SAE by using a large population-based study. Psychosocial interventions are known to reduce depressive symptoms in women and men with SAE [61]. However, when sexual assault victims undergo examination by a physician to preserve evidence and document injuries, healthcare providers should assess the substance use in women, including tobacco and cannabis, and in men, including tobacco and alcohol. This assessment should be conducted during follow-up care to determine whether the use is excessive and whether additional support from professionals such as psychiatrists and psychotherapists is necessary to improve the overall health and well-being of the patient. Future research should continue to examine the relationship between heavy substance use and depression in sexual assault victims using gender-specific prospective data. It may also be helpful to consider data from clinical encounters or registries, and to follow patients during and after treatment for sexual assault.

## Conclusions

First, our results suggest that SAE is a serious public health problem in Germany. Second, our results imply that heavy substance use can play a critical role in the relationship between SAE and depression. Third, there is a reciprocal relationship between heavy substance use and depression, suggesting that addressing one may have implications for the other. Finally, our findings imply that substance use and depression and their relationship should always be mentioned clinically in individuals who have experienced sexual assault.

Public health and policy interventions could reduce the risk of sexual assault, and in turn, heavy substance use and depression. Strategies needed to achieve these goals include separate prevention programs for sexual assault and heavy substance use at the individual, community, and societal levels. On the other hand, it is imperative that clinicians and therapists implement timely interventions to prevent the onset of addiction and depressive disorders following sexual assault and rape.

## Electronic supplementary material

Below is the link to the electronic supplementary material.

Supplementary Material 1

Supplementary Material 2

Supplementary Material 3

Supplementary Material 4

## Data Availability

The data associated with this study have not been deposited into a publicly accessible repository. The data supporting the results of this study are available from the University Medical Center Hamburg-Eppendorf; however, there are restrictions on their availability. These data were used under license for the current study and are therefore not publicly accessible. Nevertheless, the data will be made available from the corresponding author (Matthias Belau/e-mail: m.belau@uke.de) upon reasonable request and with the permission of the University Medical Center Hamburg-Eppendorf.

## Abbreviations

CI: Confidence interval
GeSiD: German Health and Sexuality Survey
NDE: Natural direct effect
NIE: Natural indirect effect
PM: Proportion mediated
SAE: Sexual assault experience
TE: Total effect
Q1: First quartile
Q3: Third quartile
