# Supplementary material for: Sexual assault experience, depression, and heavy substance use among German adults: an exploratory mediation analysis
Source: BMC Public Health. 2025 Mar 10;25:935. doi: 10.1186/s12889-025-22117-4 (PMC11892163; doi:10.1186/s12889-025-22117-4)
Supplement: Supplementary file 1 — Supplementary Material 1 [file 12889_2025_22117_MOESM1_ESM.docx]

**Supplementary Material S1**

**Table S1:** Prevalence of depression by
sociodemographic, sexual assault experience, and heavy substance use characteristics (N=4,867)

|  | | | **Depression** | | | | | | |
| --- | --- | --- | --- | --- | --- | --- | --- | --- | --- |
|  |  |  | **Women**  n=2,563 | | |  | **Men**  n=2,304 | | |
|  |  |  | **%** | **95% CI** | |  | **%** | **95% CI** | |
|  |  |  |  | **Lower** | **Upper** |  |  | **Lower** | **Upper** |
| **Age group** | | |  |  |  |  |  |  |  |
|  | | 18-25 | 11.3 | 8.2 | 15.3 |  | 3.3 | 1.9 | 5.9 |
|  |  | 26-35 | 6.9 | 4.9 | 9.6 |  | 4.9 | 3.0 | 7.7 |
|  |  | 36-45 | 10.6 | 7.4 | 14.8 |  | 7.8 | 5.0 | 11.9 |
|  |  | 46-55 | 10.1 | 7.0 | 14.3 |  | 6.9 | 4.5 | 10.4 |
|  |  | 56-65 | 16.2 | 12.6 | 20.7 |  | 6.8 | 4.4 | 10.4 |
|  |  | 66-75 | 8.8 | 5.7 | 13.5 |  | 2.7 | 1.2 | 6.1 |
| **Education** | | |  |  |  |  |  |  |  |
|  | | High | 9.7 | 7.8 | 12.0 |  | 6.3 | 4.9 | 8.2 |
|  |  | Medium | 9.7 | 7.7 | 12.3 |  | 6.1 | 4.3 | 8.6 |
|  |  | Low | 13.4 | 10.0 | 17.8 |  | 4.6 | 3.0 | 7.1 |
| **SAE** (lifetime event) | | |  |  |  |  |  |  |  |
|  | | No | 8.6 | 7.2 | 10.1 |  | 5.4 | 4.4 | 6.6 |
|  |  | Yes | 24.1 | 19.5 | 29.5 |  | 17.4 | 7.8 | 34.6 |
| **SAE** (childhood event) | | |  |  |  |  |  |  |  |
|  | No | | 8.5 | 7.2 | 10.1 |  | 5.3 | 4.3 | 6.5 |
|  | Yes | | 36.0 | 24.5 | 49.3 |  | 7.9 | 0.6 | 54.3 |
| **Hazardous alcohol use** | | |  |  |  |  |  |  |  |
|  | | No | 10.8 | 9.4 | 12.4 |  | 5.6 | 4.6 | 6.9 |
|  |  | Yes | 16.3 | 8.9 | 27.9 |  | 14.8 | 5.4 | 34.9 |
| **Heavy tobacco use** | | |  |  |  |  |  |  |  |
|  | | No | 10.0 | 8.7 | 11.5 |  | 5.0 | 4.0 | 6.3 |
|  |  | Yes | 20.2 | 13.6 | 29.0 |  | 9.3 | 5.7 | 14.7 |
| **Frequent cannabis use** | | |  |  |  |  |  |  |  |
|  | | No | 10.3 | 9.0 | 11.9 |  | 5.5 | 4.5 | 6.7 |
|  |  | Yes | 42.6 | 22.2 | 65.9 |  | 11.5 | 5.3 | 23.1 |
| %: quantity (weighted), CI: confidence interval (weighted), n: quantity (unweighted), SAE: sexual assault experience | | | | | | | | | |
