# Supplementary material for: Sexual assault experience, depression, and heavy substance use among German adults: an exploratory mediation analysis
Source: BMC Public Health. 2025 Mar 10;25:935. doi: 10.1186/s12889-025-22117-4 (PMC11892163; doi:10.1186/s12889-025-22117-4)
Supplement: Supplementary file 2 — Supplementary Material 2 [file 12889_2025_22117_MOESM2_ESM.docx]

**Supplementary Material S2**

**Table S2:** Prevalence of heavy substance use by sociodemographic, sexual assault experience, and depression characteristics (N=4,867)

|  | | | **Hazardous alcohol use** | | | | | | |  | **Heavy tobacco use** | | | | | | |  | **Frequent cannabis use** | | | | | | |
| --- | --- | --- | --- | --- | --- | --- | --- | --- | --- | --- | --- | --- | --- | --- | --- | --- | --- | --- | --- | --- | --- | --- | --- | --- | --- |
|  |  |  | **Women**  n=2,563 | | |  | **Men**  n=2,304 | | |  | **Women**  n=2,563 | | |  | **Men**  n=2,304 | | |  | **Women**  n=2,563 | | |  | **Men**  n=2,304 | | |
|  |  |  | **%** | **95% CI** | |  | **%** | **95% CI** | |  | **%** | **95% CI** | |  | **%** | **95% CI** | |  | **%** | **95% CI** | |  | **%** | **95% CI** | |
|  |  |  |  | **Lower** | **Upper** |  |  | **Lower** | **Upper** |  |  | **Lower** | **Upper** |  |  | **Lower** | **Upper** |  |  | **Lower** | **Upper** |  |  | **Lower** | **Upper** |
| **Age group** | | |  |  |  |  |  |  |  |  |  |  |  |  |  |  |  |  |  |  |  |  |  |  |  |
|  | | 18-25 | 2.9 | 1.4 | 5.8 |  | 2.2 | 1.1 | 4.1 |  | 2.5 | 1.1 | 5.9 |  | 7.0 | 4.1 | 11.5 |  | 2.6 | 1.2 | 5.4 |  | 6.0 | 3.9 | 9.0 |
|  |  | 26-35 | 0.2 | 0.1 | 1.1 |  | 0.9 | 0.3 | 2.1 |  | 6.5 | 4.4 | 9.7 |  | 11.4 | 8.8 | 14.7 |  | 1.5 | 0.8 | 2.8 |  | 5.7 | 3.9 | 8.2 |
|  |  | 36-45 | 0.7 | 0.1 | 3.2 |  | 0.2 | 0.1 | 1.9 |  | 7.5 | 4.8 | 11.4 |  | 13.2 | 9.6 | 17.9 |  | 1.3 | 0.4 | 4.0 |  | 2.2 | 1.0 | 4.6 |
|  |  | 46-55 | 0.1 | 0.1 | 0.8 |  | 0.8 | 0.2 | 2.9 |  | 9.3 | 6.5 | 13.1 |  | 17.2 | 13.2 | 22.1 |  | 0.5 | 0.2 | 2.1 |  | 1.4 | 0.6 | 3.3 |
|  |  | 56-65 | 0.9 | 0.3 | 2.9 |  | 0.8 | 0.2 | 2.6 |  | 12.0 | 8.9 | 16.0 |  | 14.8 | 10.9 | 19.8 |  | 0.2 | 0.1 | 0.7 |  | 0.4 | 0.1 | 1.9 |
|  |  | 66-75 | - | - | - |  | 0.8 | 0.1 | 6.0 |  | 5.8 | 3.1 | 10.3 |  | 5.4 | 2.9 | 9.6 |  | 0.8 | 0.1 | 5.9 |  | 0.2 | 0.1 | 1.8 |
| **Education** | | |  |  |  |  |  |  |  |  |  |  |  |  |  |  |  |  |  |  |  |  |  |  |  |
|  | | High | 0.4 | 0.2 | 0.9 |  | 0.7 | 0.4 | 1.4 |  | 3.6 | 2.4 | 5.2 |  | 5.6 | 4.2 | 7.3 |  | 0.3 | 0.1 | 1.4 |  | 2.8 | 1.7 | 4.6 |
|  |  | Medium | 0.5 | 0.2 | 1.4 |  | 1.1 | 0.5 | 2.6 |  | 7.7 | 5.9 | 10.0 |  | 15.2 | 12.2 | 18.7 |  | 1.3 | 0.6 | 2.8 |  | 2.7 | 1.7 | 4.4 |
|  |  | Low | 1.2 | 0.5 | 2.9 |  | 0.9 | 0.3 | 2.2 |  | 13.3 | 10.0 | 17.4 |  | 17.0 | 13.6 | 21.1 |  | 1.2 | 0.7 | 2.2 |  | 2.2 | 1.5 | 3.3 |
| **SAE** (lifetime event) | | |  |  |  |  |  |  |  |  |  |  |  |  |  |  |  |  |  |  |  |  |  |  |  |
|  | | No | 0.6 | 0.3 | 1.1 |  | 0.8 | 0.5 | 1.3 |  | 7.2 | 6.0 | 8.7 |  | 12.0 | 10.3 | 13.9 |  | 0.7 | 0.4 | 1.2 |  | 2.6 | 2.0 | 3.4 |
|  |  | Yes | 1.1 | 0.3 | 3.8 |  | 4.1 | 0.9 | 16.2 |  | 11.2 | 7.6 | 16.3 |  | 23.3 | 12.9 | 38.5 |  | 3.0 | 1.9 | 4.9 |  | 1.1 | 0.1 | 6.9 |
| **SAE** (childhood event) | | |  |  |  |  |  |  |  |  |  |  |  |  |  |  |  |  |  |  |  |  |  |  |  |
|  | No | | 0.6 | 0.3 | 1.1 |  | 0.8 | 0.5 | 1.3 |  | 7.2 | 6.0 | 8.7 |  | 12.0 | 10.3 | 13.9 |  | 0.7 | 0.4 | 1.2 |  | 2.6 | 2.0 | 3.4 |
|  | Yes | | - | - | - |  | 13.2 | 2.2 | 51.0 |  | 13.7 | 6.9 | 25.2 |  | 41.8 | 18.5 | 69.4 |  | 2.7 | 1.1 | 6.6 |  | 0.0 | 0.0 | 0.0 |
| **Depression** | | |  |  |  |  |  |  |  |  |  |  |  |  |  |  |  |  |  |  |  |  |  |  |  |
|  | | No | 0.7 | 0.4 | 1.2 |  | 0.8 | 0.5 | 1.4 |  | 6.9 | 5.8 | 8.3 |  | 11.8 | 10.1 | 13.8 |  | 0.6 | 0.4 | 1.1 |  | 2.4 | 1.8 | 3.1 |
|  |  | Yes | 0.4 | 0.1 | 1.9 |  | 1.8 | 0.4 | 7.8 |  | 14.6 | 9.8 | 21.2 |  | 20.5 | 13.3 | 30.1 |  | 4.2 | 2.1 | 8.3 |  | 5.2 | 2.4 | 11.0 |
| %: quantity (weighted), CI: confidence interval (weighted), n: quantity (unweighted), SAE: sexual assault experience | | | | | | | | | | | | | | | | | | | | | | | | | |
