# Supplementary material for: Sexual assault experience, depression, and heavy substance use among German adults: an exploratory mediation analysis
Source: BMC Public Health. 2025 Mar 10;25:935. doi: 10.1186/s12889-025-22117-4 (PMC11892163; doi:10.1186/s12889-025-22117-4)
Supplement: Supplementary file 3 — Supplementary Material 3 [file 12889_2025_22117_MOESM3_ESM.docx]

**Supplementary Material S3**

**Sensitivity Analysis**

**Table S3:** Mediation analyses between sexual assault experienced more than once (lifetime, childhood event) and depression by sex, adjusted for age, and education (N=4,632)

|  | |  | **Lifetime event** ^a^ | | | | | | |  | **Childhood event** ^b^ | | | | | | |
| --- | --- | --- | --- | --- | --- | --- | --- | --- | --- | --- | --- | --- | --- | --- | --- | --- | --- |
| **Depression** |  |  | **Women** | | |  | **Men** | | |  | **Women** | | |  | **Men** | | |
|  |  |  | **OR** | **95% BCI** | |  | **OR** |  |  |  | **OR** | **95% BCI** | |  | **OR** | **95% BCI** | |
|  |  |  |  | **Lower** | **Upper** |  |  | **Lower** | **Upper** |  |  | **Lower** | **Upper** |  |  | **Lower** | **Upper** |
| *Mediator* |  |  |  |  |  |  |  |  |  |  |  |  |  |  |  |  |  |
| Hazardous alcohol use | Total effect |  | 5.44 | 3.56 | 8.30 |  | 3.70 | 0.59 | 11.29 |  | 6.25 | 3.12 | 13.13 |  | 2.23 | 1.30 | 13.06 |
|  | Natural direct effect |  | 5.44 | 3.56 | 8.30 |  | 3.51 | 0.59 | 10.35 |  | 6.25 | 3.12 | 13.13 |  | 1.86 | 1.07 | 7.44 |
|  | Natural indirect effect |  | 1.00 | 1.00 | 1.00 |  | 1.05 | 0.95 | 1.50 |  | 1.00 | 1.00 | 1.00 |  | 1.20 | 0.88 | 2.41 |
| *Mediator* |  |  |  |  |  |  |  |  |  |  |  |  |  |  |  |  |  |
| Heavy tobacco use | Total effect |  | 5.74 | 3.73 | 8.93 |  | 3.63 | 0.52 | 10.88 |  | 6.59 | 3.35 | 13.52 |  | 2.18 | 1.27 | 11.45 |
|  | Natural direct effect |  | 5.61 | 3.62 | 8.73 |  | 3.29 | 0.43 | 9.82 |  | 6.41 | 3.17 | 13.39 |  | 1.75 | 1.02 | 8.98 |
|  | Natural indirect effect |  | 1.02 | 0.99 | 1.08 |  | 1.10 | 0.99 | 1.39 |  | 1.02 | 0.99 | 1.11 |  | 1.24 | 1.02 | 1.80 |
| *Mediator* |  |  |  |  |  |  |  |  |  |  |  |  |  |  |  |  |  |
| Frequent cannabis use | Total effect |  | 7.80 | 4.15 | 12.87 |  | 3.80 | 0.49 | 10.76 |  | 8.55 | 3.48 | 16.10 |  | 2.34 | 1.40 | 12.63 |
|  | Natural direct effect |  | 5.57 | 3.66 | 8.57 |  | 3.80 | 0.49 | 10.76 |  | 6.59 | 3.10 | 12.40 |  | 2.34 | 1.40 | 12.63 |
|  | Natural indirect effect |  | 1.39 | 1.07 | 3.12 |  | 1.00 | 1.00 | 1.00 |  | 1.29 | 0.97 | 2.00 |  | 1.00 | 1.00 | 1.00 |
| OR, odds ratio; BCI, confidence interval from 1,000 bootstrap samples, ^a^ Sample size ranged from 2,251 to 2,360 in women and from 2,196 to 2,263 in men, ^b^ Sample size ranged from 2,134 to 2,239 in women and from 2,181 to 2,247 in men | | | | | | | | | | | | | | | | | |
