# Supplementary material for: Sexual assault experience, depression, and heavy substance use among German adults: an exploratory mediation analysis
Source: BMC Public Health. 2025 Mar 10;25:935. doi: 10.1186/s12889-025-22117-4 (PMC11892163; doi:10.1186/s12889-025-22117-4)
Supplement: Supplementary file 4 — Supplementary Material 4 [file 12889_2025_22117_MOESM4_ESM.docx]

**Supplementary Material S4**

**Sensitivity Analysis**

**Table S4:** Mediation analyses between sexual assault experienced more than once (lifetime, childhood event) and heavy substance use by sex, adjusted for age, and education (N=4,632)

|  | |  | **Lifetime event** ^a^ | | | | | | |  | **Childhood event** ^b^ | | | | | | |
| --- | --- | --- | --- | --- | --- | --- | --- | --- | --- | --- | --- | --- | --- | --- | --- | --- | --- |
| **Hazardous alcohol use** |  |  | **Women** | | |  | **Men** | | |  | **Women** | | |  | **Men** | | |
|  |  |  | **OR** | **95% BCI** | |  | **OR** |  |  |  | **OR** | **95% BCI** | |  | **OR** | **95% BCI** | |
|  |  |  |  | **Lower** | **Upper** |  |  | **Lower** | **Upper** |  |  | **Lower** | **Upper** |  |  | **Lower** | **Upper** |
| *Mediator* |  |  |  |  |  |  |  |  |  |  |  |  |  |  |  |  |  |
| Depression | Total effect |  | 0.88 | 0.82 | 1.30 |  | 11.53 | 3.21 | 63.58 |  | 0.84 | 0.73 | 1.38 |  | 32.0 | 3.56 | 100.43 |
|  | Natural direct effect |  | 1.00 | 1.00 | 1.00 |  | 10.05 | 3.16 | 42.58 |  | 1.00 | 1.00 | 1.00 |  | 29.0 | 7.02 | 90.82 |
|  | Natural indirect effect |  | 0.88 | 0.82 | 1.30 |  | 1.14 | 0.91 | 1.79 |  | 0.84 | 0.73 | 1.38 |  | 1.10 | 0.99 | 2.17 |
| **Heavy tobacco use** |  |  | **Women** | | |  | **Men** | | |  | **Women** | | |  | **Men** | | |
|  |  |  | **OR** | **95% BCI** | |  | **OR** | **95% BCI** | |  | **OR** | **95% BCI** | |  | **OR** | **95% BCI** | |
|  |  |  |  | **Lower** | **Upper** |  |  | **Lower** | **Upper** |  |  | **Lower** | **Upper** |  |  | **Lower** | **Upper** |
| *Mediator* |  |  |  |  |  |  |  |  |  |  |  |  |  |  |  |  |  |
| Depression | Total effect |  | 1.94 | 1.09 | 3.39 |  | 3.49 | 1.12 | 9.37 |  | 2.69 | 0.91 | 5.60 |  | 6.88 | 1.97 | 25.38 |
|  | Natural direct effect |  | 1.60 | 0.88 | 2.85 |  | 2.97 | 0.87 | 7.94 |  | 2.20 | 0.66 | 4.89 |  | 6.25 | 1.81 | 23.02 |
|  | Natural indirect effect |  | 1.21 | 1.03 | 1.52 |  | 1.17 | 0.95 | 1.60 |  | 1.21 | 0.98 | 1.62 |  | 1.09 | 0.98 | 1.37 |
| **Frequent cannabis use** |  |  | **Women** | | |  | **Men** | | |  | **Women** | | |  | **Men** | | |
|  |  |  | **OR** | **95% BCI** | |  | **OR** | **95% BCI** | |  | **OR** | **95% BCI** | |  | **OR** | **95% BCI** | |
|  |  |  |  | **Lower** | **Upper** |  |  | **Lower** | **Upper** |  |  | **Lower** | **Upper** |  |  | **Lower** | **Upper** |
| *Mediator* |  |  |  |  |  |  |  |  |  |  |  |  |  |  |  |  |  |
| Depression | Total effect |  | 4.04 | 1.02 | 12.28 |  | 1.29 | 0.89 | 2.08 |  | 3.57 | 0.71 | 17.85 |  | 1.15 | 0.96 | 2.06 |
|  | Natural direct effect |  | 2.34 | 0.54 | 9.24 |  | 1.00 | 1.00 | 1.00 |  | 1.87 | 0.30 | 16.74 |  | 1.00 | 1.00 | 1.00 |
|  | Natural indirect effect |  | 1.72 | 1.07 | 3.01 |  | 1.29 | 0.89 | 2.08 |  | 1.90 | 0.85 | 3.32 |  | 1.15 | 0.96 | 2.06 |
| OR, odds ratio; BCI, confidence interval from 1,000 bootstrap samples, ^a^ Sample size was 2,364 in women and 2,268 in men, ^b^ Sample size was 2,242 in women and 2,252 in men | | | | | | | | | | | | | | | | | |
